# Inclusion of the fitness sharing technique in an evolutionary algorithm to analyze the fitness landscape of the genetic code adaptability

**DOI:** 10.1186/s12859-017-1608-x

**Published:** 2017-03-27

**Authors:** José Santos, Ángel Monteagudo

**Affiliations:** Department of Computer Science, University of A Coruña, Campus de Elviña s/n, A Coruña, 15071 Spain

**Keywords:** Genetic code adaptability, Evolutionary computing, Genetic algorithm, Fitness sharing

## Abstract

**Background:**

The canonical code, although prevailing in complex genomes, is not universal. It was shown the canonical genetic code superior robustness compared to random codes, but it is not clearly determined how it evolved towards its current form.

The error minimization theory considers the minimization of point mutation adverse effect as the main selection factor in the evolution of the code. We have used simulated evolution in a computer to search for optimized codes, which helps to obtain information about the optimization level of the canonical code in its evolution.

A genetic algorithm searches for efficient codes in a fitness landscape that corresponds with the adaptability of possible hypothetical genetic codes. The lower the effects of errors or mutations in the codon bases of a hypothetical code, the more efficient or optimal is that code.

The inclusion of the fitness sharing technique in the evolutionary algorithm allows the extent to which the canonical genetic code is in an area corresponding to a deep local minimum to be easily determined, even in the high dimensional spaces considered.

**Results:**

The analyses show that the canonical code is not in a deep local minimum and that the fitness landscape is not a multimodal fitness landscape with deep and separated peaks. Moreover, the canonical code is clearly far away from the areas of higher fitness in the landscape.

**Conclusions:**

Given the non-presence of deep local minima in the landscape, although the code could evolve and different forces could shape its structure, the fitness landscape nature considered in the error minimization theory does not explain why the canonical code ended its evolution in a location which is not an area of a localized deep minimum of the huge fitness landscape.

## Background

The canonical or standard genetic code is redundant since the 64 possible codons encode only 21 labels, the 20 amino acids present in proteins and the “stop” signal that defines the end of the protein translation process. The canonical code, although prevailing in complex genomes, is not universal. The existence of other different codes, like the one of mitochondrial DNA, altered the “frozen accident”, as coined by Crick [1], so extensive research has been performed in order to analyze the reasons behind the establishment of the canonical genetic code.

There are three main theories about the genetic code organization and development, which could have influenced the canonical code organization. The stereochemical theory states that the stereochemical interactions between bases and amino acids influenced the primordial code, probably in the RNA World or earlier [2, 3]. Thus, the physicochemical affinity between amino acids and the cognate codons determined the codon assignments [4]. The second one is the coevolution theory [5], which maintains that at an early stage of the genetic code development few precursor amino acids were encoded. The other amino acids (product amino acids) developed biosynthetically from such precursor amino acids and these initial amino acids passed part or their whole codon domain to their biosynthetically produced amino acids. Finally, the error minimization theory or physicochemical theory considers the minimization of point mutation adverse effects as the main selection factor in the evolution of the code [4, 6, 7].

According to this last theory, the genetic code structure evolved to maximize its robustness, that is, to minimize the consequences of code mutations on the function of the encoded proteins [4]. In favor of this theory is the fact that similar codons encode amino acids with similar chemical properties: the codons that share two bases tend to code amino acids with similar hydrophobicity.

In this last alternative, a huge number of alternative “genetic codes” are possible. The number of possible alternative codes is 1.4·10^70^ [8], when each amino acid is coded by 6 codons at maximun (like the canonical code case). If only permutations of the amino acids encoded in the 20 codon sets of the canonical code are allowed, there are 20! (2.43·10^18^) possible codes. This alternative, which maintains the canonical codon set structure, is the most used in the studies regarding the error minimization theory. Finally, more than 1.51·10^84^ codes can be defined, when no restrictions in the associations between the 64 codons and the 21 meanings are considered [9].

In this error minimization theory, the efficiency or optimality of a code is defined taking into consideration the possible errors or mutations in the codon letters. Typically, all the point mutations in the codons are applied to quantify the change between the encoded amino acids before and after each point mutation. That change is measured taking into account an amino acid property, the polar requirement property being the one most used. Once such changes are averaged over all the possible mutations, the lower that error value of a code the more efficient or optimal is the corresponding code, since it means smaller phenotypic changes in the encoded proteins when mutations happen.

Moreover, two different analyses were considered to assess the optimality or adaptability of the canonical genetic code. In the first one, named “statistical analysis”, many randomly generated codes are defined and then the probability of more efficient random codes with respect to the canonical one is quantified. The lower this probability the more optimized is the canonical code. According the authors that follow this analysis [2, 10–12], the pattern of codon assignments of the canonical code appears nearly optimal.

For example, in this statistical approach, Freeland and Hurst [10] used alternative codes with the same codon set block structure of the canonical code. In an ample sample of 1,000,000 possible alternative codes only 114 codes were more efficient than the canonical code (the criteria for defining such hypothetical codes are summarized in the “Methods” section, together with the measure to quantify code optimality). This low number of random better codes allows the authors to state that the canonical genetic code evolved under the selection of the error minimization. Moreover, when the authors weighted transition mutations differently from transversion mutations, only 1 in every million randomly alternative codes was better than the canonical genetic code, as the article title states [10].

Extensions of these initial works include the analyses by Gilis et al. [13], where the authors considered the role of amino-acid frequencies in the efficiency of the canonical genetic code, and the work by Torabi et al. [14] who have experimented with the role of aminoacyl-tRNA synthetases in decreasing the effects of mistranslations in the code evolution. Along this line, Zhu et al. [15] also took into consideration the codon usage of individual species in the code optimization for error minimization and Marquez et al. [16] checked whether organisms optimize the genetic code at the same time that the codon usage.

The second approach to assess code efficiency is the “engineering approach” [17, 18]. First, the most efficient code is found, typically by a computational search method. Afterwards, the error value of the canonical code is compared with the average error value of randomly generated codes and the error value of the best obtained code. The relative position of the canonical code error value with respect to the others provides a measure of the optimization level of the canonical code. The results with this approach show that the canonical code is not so close to the optimal as the statistical approach claims. The authors in [17, 19] have discussed and debated these two alternatives.

For example, in this engineering approach, according to Di Giulio [20], the canonical genetic code achieved 72.7% minimization of polarity distance when comparing the error value of the canonical code with the error values of random codes defined with the same codon set structure of the canonical one and with the value of the best possible code (The same methodology explained in the “Methods” section was used to assess the adaptability level of a possible code). The best code (necessary in the comparison) was obtained by Di Giulio [20] using simulated annealing. Novozhilov et al. [21], in order to investigate the optimization of codes for the maximum attainable robustness, used also a greedy minimization algorithm for searching better alternative codes. Their search method employed swaps of four-codon or two-codon series, in alternative codes defined with the same block structure and the same degeneracy degree of the canonical code. According to the authors’ results, the canonical code is much closer to its local fitness minimum than the majority of the random codes with similar robustness to the canonical one.

Following other alternatives, Gardini et al. [22] focused their analysis on searching clues for the canonical code robustness. Given the dependence of protein function on the tridimensional structure conformation, they took into consideration the function of amino acids in their specific structural environment, analyzing the role of each amino acid in inner or outer regions of the protein structure. In their work [22], the Protein Data Bank ample information was used through a structural bioinformatics approach to search for unambiguous clues of the rationale of the canonical genetic code in assigning from one to six different codons for the different amino acids. For example, Leu and Arg offer a clear clue, since both appear in the canonical code with six assigned codons, therefore with a high protection from translational errors, and those also appear as the most abundant amino acids in protein-protein and protein-nucleic acid interactions.

Our work is focused on the error minimization theory. Previously, we have used a Genetic Algorithm (GA), as a search method for finding better adapted codes than the canonical one [23]. The GA provides a global search in the fitness landscape associated with the adaptability of possible hypothetical codes, allowing to obtain clues about the difficulty to obtain better optimized codes. Moreover, we also employed a model of alternative codes which reflects the known codon reassignments [24]. In line with the engineering approach, our results with simulated evolution revealed that the canonical code is far from the best possible optimized codes. Extending our previous work [23], Oliveira et al. [25] also proposed a multiobjective approach since two or more objectives were simultaneously optimized. They used as objectives code robustness against mutations, considering the changes in the polar requirement of amino acids (objective 1), and code robustness with respect to the hydropathy index or molecular volume changes under mutations of possible hypothetical codes (objective 2). The comparison between the evolution with only one objective and the use of a multiobjective evolutionary algorithm shown that the multiobjective alternative obtains optimized solutions closer to the canonical genetic code. Moreover, Oliveira and Tinós [26] proposed a function which uses entropy with the aim to increase the variability in the number of codons assigned to amino acids. That is, the effects against mutations of a code should be minimized while its entropy should be maximized. With this consideration, their results also indicate that the canonical genetic code is slightly better optimized with respect to not using the entropy term. Also, BlaŻej et al. [27], inspired by our work with the adapted GA [23], analyzed the effectiveness of using various combinations of mutation and crossover probabilities under three models of the genetic code, assuming different restrictions on its structure.

In evolutionary computing, the so-called “fitness sharing technique” [28] is a “niching” method that allows the evolutionary algorithm search to be simultaneous performed in different areas (niches) corresponding to different local (or global) optima, that is, the technique permits the identification and localization of multiple optima in the search space. It should be noted that the optimization of the code adaptability turns into a minimization problem, where the “code fitness” or adaptability is inversely related to the error cost of a code (the more robust a code is against base mutations, the larger the fitness and the lower the error cost, “Methods” section). In the present work, the fitness sharing technique is introduced into the evolutionary algorithm, which allows the extent to which the canonical genetic code is in an area corresponding to a deep local minimum to be easily determined, even in the high dimensional spaces considered.

It is clearly not difficult to discern whether the canonical code is in a local minimum regarding the fitness landscape associated with the code adaptability. It is only necessary to consider the adaptability level in its neighborhood, that is, inspecting the fitness landscape in its close neighborhood. For example, Novozhilov et al. found that “The standard genetic code appears to be a point on an evolutionary trajectory from a random point (code) about half the way to the summit of the local peak” [21]. The fitness landscape is clearly rugged [21], but the question to answer in this paper is the following: is the standard genetic code in an area corresponding to a deep and separated local peak in the vast fitness landscape? Moreover, another related aspect is about the possible multimodal nature of the fitness landscape. That is, does the landscape, even with its rugged nature with many non-deep peaks, present localized areas of high fitness (adaptability) separated by low fitness barriers?

The answers to the questions are relevant since many previous works and authors discussed the possibility of the location of the standard genetic code in a local minimum (or close to it), regarding error cost, to explain its non-optimum adaptability. That information about the general surface of the fitness landscape could provide clues about the difficulty of the possible evolution of the canonical genetic code. Since an exhaustive search of the landscape is not possible, evolutionary computing was used to search in the promising areas of the fitness landscape, incorporating the aforementioned useful technique in evolutionary algorithms (fitness sharing) in order to obtain clues about the multimodal nature of the fitness landscape and the relative depth of its peaks.

In the rest of the paper the “Methods” section details the definitions of the alternative codes, their encoding in the GA population, the GA operators, the fitness definition in the landscape of such alternative codes, the fitness sharing technique as well as the measures used to quantify the canonical genetic code adaptability level. The “Results” section expounds the experiment results when the fitness sharing technique is introduced in the GA. Finally, the last sections present a discussion of the results and final conclusions.

## Methods

### Generation of variant genetic codes

Two possibilities of hypothetical codes were considered. The first possibility reflects the current genetic code translation table and is the most used in previous works [10, 12, 13]. When hypothetical codes were generated, two restrictions were considered:

1. The codon space (64 codons) was divided into 21 nonoverlapping sets of codons observed in the standard genetic code, each set comprising all codons specifying a particular amino acid in the standard code. Twenty sets correspond to the amino acids and one set to the 3 stop codons.
2. Each alternative code is formed by randomly assigning each of the 20 amino acids to one of these sets. The three stop codons remain invariant in position for all the alternative codes. Moreover, these three codons are the same stop codons of the standard genetic code (UAA, UAG and UGA).

This conservative restriction, which maintains the pattern of synonymous coding found with the standard genetic code, controls, as indicated by Freeland [29], possible biochemical restrictions on code variation and the level of redundancy inherent in the canonical code [11]; or, as stated by Novozhilov et al. [21], “The premise behind this choice is that the block structure of the code is a direct, mechanistic consequence of the mode of interaction between the ribosome, mRNA, and the cognate tRNA”.

Although these authors (Novozhilov et al. [21]) indicate that codes with different block structures are not unviable or impossible “but they are likely to be substantially less fit than those with the canonical block structure”, we used a second possibility with the definition of hypothetical codes with only one restriction: three codons for the stop signal are only imposed. The aim of the introduction of this last possibility, also used by Di Giulio et al. [18], is a comparison between the restrictive and non-restrictive hypothetical codes in terms of optimal values that can be obtained and in terms of location of the canonical genetic code in the fitness landscape associated with these two genetic code models.

### Genetic algorithm adapted to the problem

Evolutionary computing was used for searching for optimal codes. A classical GA [30] with ad hoc operators for our problem was implemented [23]. The genetic population encodes possible hypothetical codes, whereas the fitness function is associated with the robustness against base mutations in each code. These aspects are detailed in the following subsections.

#### Encoding

Each individual of the genetic population must encode a hypothetical code. In our solution, in the case of non-restrictive codes, each individual has 64 positions, which correspond to the 64 codons, and each position encodes the particular amino acid associated with the codon (or the stop signal). As in [18], the stop signal is defined by three codons in each possible code.

In the case of restrictive codes, each individual has 20 positions, which correspond to the 20 codon sets, and each position encodes the particular amino acid associated with the codon set. In the encoding of a possible code, there is not a genotype position for the stop signal, since, as mentioned previously, the same codon set of the standard genetic code was used in all the individuals to define the stop signal.

With the non-restrictive codes, the individuals of the initial population correspond with random assignments of amino acids and the stop signal to the 64 codons, ensuring that all individuals encode, at least in one position, the 20 amino acids, in addition that three codons encode the stop signal. In the case of restrictive codes, the initial individuals are defined by random assignments between the 20 amino acids and the 20 codon sets.

#### Genetic operators

In the case of non-restrictive codes, a mutation operator and a swap operator were used. A mutation changes the amino acid encoded in each of the 64 positions, with a mutation probability, to a different one. The mutation does not operate if the amino acid to mutate is the only one in the whole code. These mutations simulate the possible errors in the transcription process from DNA to RNA and in the translation process when incorrect transfer RNAs join a given codon of the messenger RNA. From our application point of view, it is the operator that varies the number of codons associated with a particular amino acid.

**Fig. 1:**
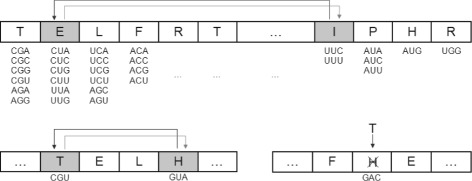
Genetic operators. *Upper part*: Encoding of a genetic code and functioning of the swap operator with the restrictive codes. *Bottom part*: Swap operator (*left*) and mutation operator (*right*) used with the unrestrictive codes

The other genetic operator is a swap operator which interchanges the contents of two genotypic positions, that is, once two positions are randomly selected, the amino acids (or stop signal) codified by the two respective codons are swapped. The bottom part of Fig. 1 shows the basic functioning of these operators.

The two operators guarantee that the 20 amino acids are always represented in the individuals. Other operators, such as the classical crossover operator, do not guarantee this important restriction.

In the restrictive codes case, as commented previously, each individual has 20 positions encoding the particular amino acid associated with a codon set present in the canonical code. As also mentioned, a fixed number of three codons are used for the stop label, which are the same used in the canonical code.

The GA only uses a swap operator with the restrictive hypothetical codes. As in the previous case, the operator interchanges the contents of two randomly selected positions (codon sets). The upper part of Fig. 1 shows the encoding of a given code and how this operator works.

Finally, as the selection operator, the tournament selection was used with both hypothetical codes. The operator selects the best individual in a window of randomly selected individuals from the population. Hence, the size of the window determines the required selective pressure. Moreover, elitism of the best individual was used; that is, this individual is retained in the next generation without changes.

#### Fitness function and optimality measure

The measure applied, for example, by Haig and Hurst [12] and Freeland and Hurst [10] to quantify the relative efficiency of any given code was used as fitness function. The measure calculates the mean squared (MS) change in an amino acid property resulting from all possible changes to each base of all the codons within a given code. Any one change is calculated as the squared difference between the property value of the amino acid coded by the original codon and the value of the amino acid coded by the new (mutated) codon. The final error is an average of the effects of all the substitutions over the whole code. Therefore, the error △ (MS) is defined as:

1 $$ \bigtriangleup^{2} = \frac{{\sum\nolimits}_{i,j}w_{i,j}\left(X_{i}-X_{j}\right)^{2}}{{\sum\nolimits}_{i,j}N_{ij}}   $$

where *N*_*ij*_ is the number of times the *i*−*th* amino acid changes into the *j*−*th* amino acid, and *X*_*i*_ is the value of the amino acid property of the *i*−*th* amino acid. *w*_*i,j*_ is a weight associated with each possible mutation in each letter, which is set as 1 in the simple MS measure when an equal transition/transversion bias is considered. The changes from and to “stop” codons are ignored, while synonymous changes (the mutated codon encodes the same amino acid) are included in the calculation. Thus, the GA works with a minimization problem, where the MS measure is the objective to minimize by the GA operators. Therefore, as commented in the introduction, the adaptability or code fitness is inversely related to this error cost measure, since the lower the MS value the better the adaptability.

Like most authors, we have used the polar requirement as the amino acid property. The property can be considered as a measure of hydrophobicity and it was introduced by Carl Woese as a measure for the polarity of an amino acid, which is defined as a partitioning coefficient of an amino acid in a water/pyrimidine system [31].

Moreover, the previous equation can take into account the relative frequencies of transition/transversions mutations as well as mistranslations in the different bases. As stated by Freeland [29], the unequal chemical similarity of the 4 nucleotides to one another means that transition errors (substitution of a purine base into another purine, or a pyrimidine into another pyrimidine, i.e., *C*⇔*T* and *A*⇔*G*) occur more frequently than transversions (interchange of pyrimidines and purines, i.e. *C,T*⇔*A,G*).

To quantify the relative frequencies of mutations, we employed the rules from [10] used to consider the empirical data, which are summarized as:

1. Mistranslation of the second base is much less frequent than the other two positions, and mistranslation of the first base is less frequent than the third base position.
2. The mistranslations at the second base appear to be almost-exclusively transitional in nature.
3. At the first base, mistranslations appear to be fairly heavily biased toward transitional errors.
4. At the third codon position, there is very little transition bias.

Table 1 summarizes the quantification of mistranslation used to weigh the relative efficiency of the three bases. The weights *w*_*i,j*_ in Eq. 1 correspond with the particular weights in Table 1. Therefore, the MS calculation takes into account those rules and, following the same terminology of Freeland and Hurst [10], we term the MS variant as tMS. For example, the MS value of the canonical code is 5.19 whereas its tMS value is 2.63.

**Table 1:** Quantification of translational errors to measure the relative efficiency of a code (tMS)

Combined weighting	First base	Second base	Third base
For transitions	1	0.5	1
For transversions	0.5	0.1	1

As commented previously, the “engineering approach” compares the standard genetic code with the best possible alternative. The approach uses a “percentage distance minimization” (p.d.m.), which determines code optimality on a linear scale, as it is calculated as the percentage in which the canonical genetic code is in relation to the randomized mean code and the most optimized code. It is therefore defined as:

2 $$ p.d.m.=\frac{\bigtriangleup_{mean}-\bigtriangleup_{code}}{\bigtriangleup_{mean}-\bigtriangleup_{low}}\cdot100   $$

where △_*mean*_ is the average error value, obtained by averaging over many random codes, and △_*low*_ is the best (or approximated) △ value.

The measure can be interpreted as the optimization level reached during genetic code evolution [20]. For example, as previously indicated, Di Giulio et al. [18] reported a p.d.m. value of 72.7% in the case of codes with only amino acid permutations in the 20 sets of codons (restrictive codes), using a simulated annealing technique for obtaining the value of the best possible code, whereas we reported a p.d.m. value of 71% [23], using a GA for searching for the best possible code.

### Fitness sharing

Fitness sharing is a classical technique in evolutionary computing for dividing the population into different subgroups according to the similarity of the individuals. In this present work this technique is incorporated into the GA. This fitness sharing concept was introduced by Holland [32] and extended, for example, by Goldberg and Richarson [28]. The shared fitness for the *i*th individual is defined as:

3 $$ f_{shared}(i)=\frac{f_{original}(i)}{{\sum\nolimits}_{j = 1}^{N}sh\left(d_{ij}\right)}   $$

where the sharing function is calculated as:

4 $$ sh(d_{ij})=\left\{ \begin{array}{lll} 1 - \left(\frac{d_{ij}}{\sigma_{share}}\right)^{\alpha} & if ~ d_{ij}<\sigma_{share}\\ 0 & otherwise \end{array}\right.   $$

being *d*_*ij*_ the distance between individuals *i* and *j*, *σ*_*share*_ the sharing radius, *N* the population size and *α* a constant called the sharing level.

In this application, the distance *d*_*ij*_ is measured by taking into account the difference in polar requirement between the amino acids encoded in the same positions by code *i* and code *j* of the population. It is defined as the root squared deviation between both codes:

5 $$ d_{ij}=\frac{\sqrt{{\sum\nolimits}_{k=1}^{L}\left(X_{ik}-X_{jk}\right)^{2}}}{Max\_RSD}   $$

where *Max*_*RSD* is the maximum root squared deviation between two possible codes, taking into account the largest and lowest polar requirement values of amino acids (13 in Asp and 4.8 in Cys). The index *k* refers to a genotype position and *L* stands for the length of the genotypes in the individuals (20 for restrictive codes and 64 for unrestrictive codes). This ensures that distances are always in the range [0,1]. This procedure is the same in both hypothetical codes, except that in the case of unrestrictive codes the genotype positions where one of the codes encodes a stop signal are ignored (as in Eq. 1). Note that this definition of distance gives more information about the closeness of two codes than a simple calculation of how many different amino acids are encoded in the same position in the two codes. For example, in this last case, a simple swap of two amino acids in a code (distance 2 regarding different encoded amino acids) can correspond to different distances regarding Eq. 5, depending on the polar requirement values of the swapped amino acids.

Finally, if the application requires the minimization of the fitness (as in our case with MS), instead of its maximization, the formula in Eq. 3 turns to be a multiplication between the two terms ($f_{shared}(i)=f_{original}(i) \cdot {\sum \nolimits }_{j = 1}^{N}sh\left (d_{ij}\right)$). Therefore, the fitness sharing technique increases the objective to minimize (MS value) in densely populated regions.

This way, fitness sharing modifies the search landscape by reducing the payoff in densely populated regions. The main drawback of the technique is its complexity, *O*(*N* ^2^), because of the calculation of inter-distances. On the contrary, the important property is that fitness sharing tends to encourage searches in unexplored regions of the space and favors the formation of stable subpopulations [33].

**Fig. 2:**
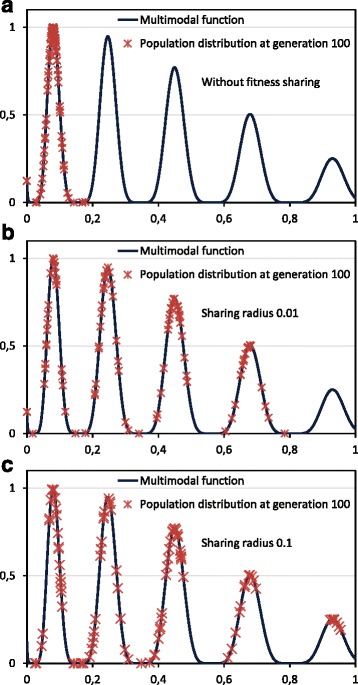
GA final population distribution to maximize a multimodal function. The function has unequally spaced peaks of nonuniform height. Final population, at generation 100, in 3 cases: **a** Without fitness sharing. **b** Using fitness sharing with *σ*_*share*_=0.01, where the population is clustered around most of the peaks of the multimodal function. **c** Using fitness sharing with *σ*_*share*_=0.1, where the population is clustered around the different peaks of the multimodal function

Figure 2 shows an example with a multimodal function commonly used as benchmark in evolutionary computing. This function [33], defined on [0,1], consists of five unequally spaced peaks of nonuniform height. Maxima are located at approximate *x* values of 0.080, 0.247, 0.451, 0.681 and 0.934. A classical genetic algorithm was run to obtain the value that maximizes that function. A simple mutation operator (the encoded parameter *x* changes to a random close value) and a tournament selection were used. The population size was 100 and the probability of mutation 0.25, running the GA across 100 generations. Without fitness sharing (Fig. 2 a), the population tends to progressively move to the highest peak, even with the low selective pressure applied (a tournament size of 3%). However, with the introduction of fitness sharing (*α*=1 in Eq. 4), now the population tends to converge at the niches of high fitness, as shown in Fig. 2 b and Fig. 2 c. Now the individuals are distributed in the peaks with a number that depends on the relative fitness of each peak. Using a higher *σ*_*share*_ (Fig. 2 c, *σ*_*share*_=0.1) the population tends to be more expanded, so it is more difficult to leave a local maximum with respect to the use of a lower *σ*_*share*_ (Fig. 2 b, *σ*_*share*_=0.01).

**Fig. 3:**
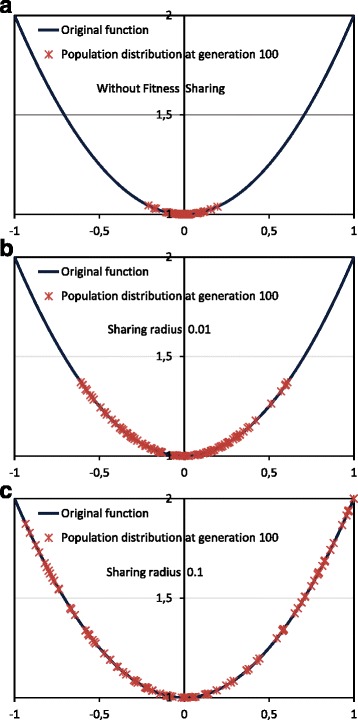
GA final population distribution to minimize the function 1+*x* ^2^. Final population, at generation 100, in 3 cases: **a** Without fitness sharing. Most of the individuals are close to the optimal value. **b** Using fitness sharing with *σ*_*share*_=0.01, with the population expanded around the minimum. **c** Using fitness sharing with *σ*_*share*_=0.1, with the population more expanded around the minimum

Figure 3 shows another example with a simple function and the same GA setup. The objective is to obtain the only minimum of the parabola. This is obviously a toy example, but the interest is to show in this function the effect of the introduction of fitness sharing that will be useful for our application. The upper part (Fig. 3 a) shows the straightforward convergence of the population towards the global minimum without the use of fitness sharing. However, the introduction of fitness sharing (*α*=1) tends to uniformly distribute the population around the minimum (Fig. 3 b), with a distribution that depends on the sharing radius (*σ*_*share*_). For example, with the largest sharing radius (*σ*_*share*_=0.1) in Fig. 3 c, the population tends to be distributed in the whole range considered for the encoded parameter *x* ([-1,1]).

That is, the interest of the fitness sharing technique is to search in all the promising areas with high fitness found in the landscape, performing a better exploration of the search space. However, the introduction of the technique also has other consequence, because it provides clues about the fitness landscape. In the first chosen example (multimodal function), the distribution of the population reveals the multimodal nature of the fitness landscape, indicating the existence of several local maxima. In the second chosen example, the population is not clustered around local minima, and therefore the population is uniformly distributed around the global minimum and according to the sharing radius. Note that this information would not be obtained without the introduction of fitness sharing. These considerations are therefore going to be taken into account in the analysis of the (high dimensional) fitness landscape when searching for hypothetical codes with optimized adaptability.

## Results

### Evolutionary algorithm setup

The implemented GA, with the incorporation of fitness sharing, was tested by searching for optimized codes, using the two code models explained in the previous section. The GA parameters for the different experiments are: population size of 1000 individuals, mutation probability of 0.01 and a swap probability of 0.5. As explained, the restrictive model only uses the swap operator to interchange the 20 amino acids among the 20 codon sets. On the contrary, both operators are used with the non-restrictive model, where the mutation allows changing the number of codons assigned to an amino acid. Tournament selection with a tournament size of 3% of the population was used, which provides a low selective pressure. These genetic parameters are the same as those used in our previous work [23], selected to provide an appropriate balance between exploration and exploitation in the GA search.

Regarding fitness sharing, the value of parameter *α* (Eq. 4) was set to 1 as it is sufficient to generate the possible clustering of the population into different niches. The sharing radius (*σ*_*share*_) was varied to observe its effect on the evolution of possible codes, whereas *d*_*ij*_ was calculated using Eq. 5 (“Methods” section), which considers the root squared deviation between code *i* and code *j* of the population, taking into account the polar requirement of the amino acids encoded in each genotype position.

### Evolution of restrictive codes

**Fig. 4:**
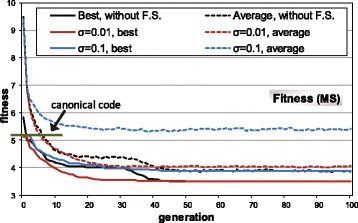
MS evolution through generations with the restrictive codes. All graphs are an average of 10 independent GA runs, without fitness sharing and fitness sharing with two values for the parameter *σ*_*share*_ (sharing radius). The graph includes the canonical code MS value for comparison (*horizontal line*)

In a first experiment, the evolutionary algorithm was used to search for optimized codes, using the model of restrictive codes. Figure 4 shows the fitness (MS) evolution through 100 generations of the genetic algorithm. Figure 4 includes the evolution without fitness sharing and with two values for the parameter sharing radius (*σ*_*share*_=0.01 and *σ*_*share*_=0.1). The graphs in Fig. 4 are an average of 10 independent runs of the GA, beginning with different random initial populations. It is not easy to determine the appropriate values to use for the parameter *σ*_*share*_, since it requires a previous knowledge about the landscape surface to easily obtain the possible clustering of the population into niches in a multimodal fitness landscape. Since we have no such a priori knowledge, we experimented with different values for the parameter *σ*_*share*_, starting with a low value (*σ*_*share*_=0.01) and also using a larger value (*σ*_*share*_=0.1) of an order of magnitude.

The evolution without fitness sharing shows that it is easy to discover better adapted codes than the canonical one. In about 50 generations, an average best value of *MS*=3.50 is obtained (by averaging the best values of the 10 independent runs). The p.d.m. value is 71%, taking into account the MS value of the canonical code (*MS*=5.19), the average value of the random codes at the initial generation of the GA and the best value in all the GA runs [23]. Even the average fitness of the population is lower with respect to the MS value of the canonical code. This shows that it is very easy for the GA to discover better adapted codes with respect to the canonical code, which denotes that the canonical code can be adapted but it is clearly far from the best possible adapted code.

When fitness sharing is considered in the GA evolution with a low sharing radius (*σ*_*share*_=0.01), the fitness progression is similar but more continuous. The reason is that fitness sharing tends to maintain the individuals at least with a distance of 0.01 between them, so it is more difficult for many individuals to correspond to the same solution, as can occur without the use of fitness sharing, even with the low selective pressure applied. With a larger sharing radius (*σ*_*share*_=0.1), the evolution of better codes is logically more difficult, although best values than the canonical code are obtained. The average fitness is now greater with respect to the canonical code and it is slightly variable through the evolutionary generations. This is because when several individuals fall in the same vicinity (easier with large values of *σ*_*share*_), their fitness is penalized, and therefore in the next generation other individuals can be selected, which generates the variability in the average fitness. Table 2 summarizes the basic statistic information about the evolutions of Fig. 4, which shows the greater variability of the final results with larger values of the parameter *σ*_*share*_.

**Table 2:** Summary of statistics regarding Fig. 4. The values are an average of 10 independent runs of the GA

		Average	Standard
		final value	deviation
Without fitness sharing	Best fitness	3.50	0.01
	Average fitness	3.88	0.04
Fitness sharing, *σ*_*share*_=0.01	Best fitness	3.50	0.01
	Average fitness	4.07	0.04
Fitness sharing, *σ*_*share*_=0.1	Best fitness	3.89	0.12
	Average fitness	5.41	0.07

**Fig. 5:**
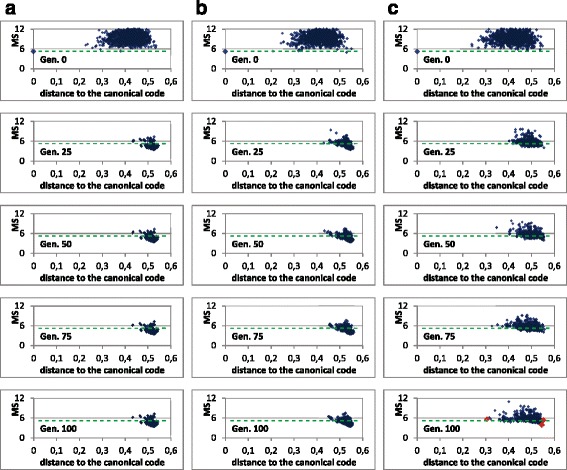
MS value of the genetic population codes vs. their distances to the canonical code. The same graph is shown in different generations of a GA run, in 3 cases with the restrictive codes: **a** without fitness sharing, **b** fitness sharing with *σ*_*share*_=0.01 and **c** fitness sharing with *σ*_*share*_=0.1. The canonical code was introduced in the initial population in the 3 cases (point with larger size). The green dashed line represents the MS value of the canonical genetic code

Nevertheless, these previous graphs of evolution of the fitness do not provide information regarding the fitness landscape in the surroundings of the canonical genetic code. We want to discern whether the canonical code is in an area corresponding to a deep local optimum in the huge search space. Figure 5 helps to visualize this.

The columns in Fig. 5 correspond with a run of the GA with three cases: In column (a) the GA was run without fitness sharing whereas in columns (b) and (c) the GA was run with fitness sharing with *σ*_*share*_=0.01 and *σ*_*share*_=0.1 respectively. In Fig. 5 the *x*-axis represents the distance of each encoded code in the population to the canonical code, whereas the *y*-axis corresponds to the MS value of each code. Therefore, Fig. 5 shows this correspondence between the MS value of each code and its corresponding distance to the canonical code in different generations of the GA.

It should be noted that if many individuals fell in the same local minimum, their distances to the canonical code would be similar as well as their MS values, that is, a cluster should be appreciated in the graph. The graphs show that the distances of the different codes of the population with respect to the canonical code vary, in most cases and after the initial generation, in the whole range between 0.4 and 0.55, which denotes that the hypothetical codes, more optimized than the canonical code in progressive generations, are far from the canonical code. This fact also indicates that the canonical code is not in an area corresponding to a deep local minimum, since all the individuals are far from it, without any individual close to it, as would occur if the canonical code was close to a deep local minimum (as explained in the “Methods” section, “Fitness sharing” subsection). The analyses with different values of *σ*_*share*_, with a sweep of *σ*_*share*_ in an ample range, between 0.001 and 0.5 (not shown in the Figures), indicate the same conclusions.

It would be useful that an individual of the GA fell close to the canonical code, since it would also provide knowledge regarding whether the canonical code is in an area corresponding to a deep local peak. Since this is very difficult with the limited number of individuals of the population, in these GA runs, the canonical code was introduced in the initial population. This allows to test whether the canonical code can “survive” in the evolutionary progress towards optimized codes. Thus, in the initial generation (with random codes except the canonical one), there is a point that corresponds to the canonical code with distance 0 and *MS*=5.19 (with larger size in Fig. 5). The other random codes present different MS values, most of them with larger values than the canonical MS value. Nevertheless, at generation 25, the canonical code has disappeared from the population (in all GA runs), indicating that it is not competitive with other hypothetical codes with better MS values at that generation. This can be obvious without fitness sharing. However, with fitness sharing, if the canonical code was in an area close to a local minimum, it would be difficult for this code to disappear from the population, as explained previously (“Methods” section. Fitness sharing), since some individuals would remain in a niche that would correspond to that area where the canonical genetic code is located. The fact that the canonical code disappears, even with a large sharing radius, is a second piece of evidence that indicates that the canonical code is not in an area corresponding to a deep local minimum.

**Fig. 6:**
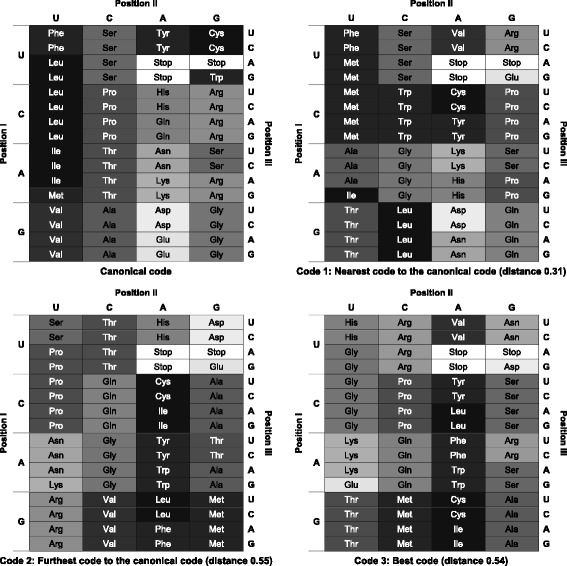
Selected evolved codes. Three codes were selected from the final population in Fig. 5.c. *Code 1*: nearest code to the canonical code. *Code 2*: furthest code to the canonical code. *Code 3*: best code. The canonical code is included for comparison. *d*_*Code*1,*Code*2_=0.40, *d*_*Code*1,*Code*3_=0.41, *d*_*Code*2,*Code*3_=0.18, *MScode*1=5.66, *MScode*2=5.49, *MScode*3=3.93

To illustrate possible evolved codes and their distances, three alternative codes were selected from the final evolved ones in Fig. 5 c (red points in the final population). Two of those codes correspond with the nearest code (*code 1*) and the furthest code (*code 2*) with respect to the canonical code (distances 0.31 and 0.55). The third one (*code 3*) is the code that has the best (minimum) MS value in that GA run of Fig. 5 c. Figure 6 shows the codes with the assignments of amino acids to the different codons. In addition, each amino acid is represented with a gray level corresponding to its polar requirement: the brighter the gray level the higher the polar requirement. The stop signal is represented in white. Moreover, Fig. 6 includes the canonical code, which helps to visualize the distance between those codes and the canonical one. For example, the differences in polar requirement (gray level) between the amino acids in the same codon positions between the canonical code and *code 1* are lower with respect to the other cases, and *code 2* and *code 3* have assignments of amino acids with similar polar requirement in the same positions. Therefore, the distance (Eq. 5) between *code 2* and *code 3* is low. However, *code 3* has the minimum MS value since it has amino acid assignments with similar polar requirement where possible mutations in the codon letters change the amino acids. For instance, *code 3* locates amino acids with very similar polar requirement in its third column (Tyr-5.4, Leu-4.9, Phe-5.0, Trp-5.2, Cys-4.8, Ile-4.9), since a mutation in the third codon letter can imply a change between such encoded amino acids.

**Fig. 7:**
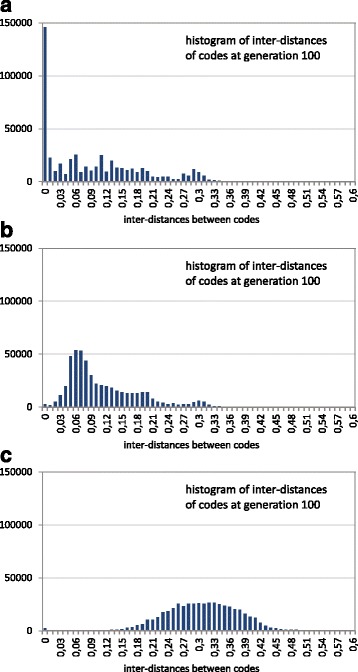
Histogram of code inter-distances of the GA final population using the restrictive codes. The histogram corresponds to the final population (generation 100) in a GA run with three cases: **a** without fitness sharing, **b** fitness sharing with *σ*_*share*_=0.01 and **c** fitness sharing with *σ*_*share*_=0.1

Finally, the previous graphs show the distances of the encoded codes with respect to a “reference point” (the canonical code), but do not show how the different codes are far away from each other. It would also be interesting to know whether the final population is clustered around a small neighborhood of the search space or it is spread in the high dimensional landscape. For this, the inter-distances of individuals of the final population were calculated, and a histogram of these distances is plotted in Fig. 7. Inter-distances close to 0 mean the possibility of a cluster (niche) of the population around a local optimum. In Fig. 7 the *x*-axis is sampled in intervals of 0.01, that is, each of the 100 intervals in the *x*-axis specifies the number of inter-distances of the population in that interval. Although the distances are normalized in the interval [0,1] (Eq. 5), it is difficult to obtain inter-distances larger than 0.6, because it would imply large changes in the polar requirement of the encoded amino acids of the codes, and many amino acids have a similar value of polar requirement.

Figure 7 shows that the codes are further away from each other as the sharing radius becomes larger. Without fitness sharing (Fig. 7 a), the individuals are closer to the best solution. In fact, at the final generation, many individuals correspond to the same solution (in this case the individuals with inter-distance 0). With the incorporation of fitness sharing and with the lower sharing radius (Fig. 7 b, *σ*_*share*_=0.01) the population is slightly more expanded through the fitness landscape, with most of the inter-distances between 0.02 and 0.32. The main difference with respect to the case without fitness sharing is that now it is more difficult that two individuals correspond to the best obtained solution. Note that the histogram shows a low height in the first interval [0,0.01) of the *x*-axis; however, this does not imply that the inter-distances correspond to equal individuals. Moreover, if it is taken into account that using fitness sharing with *σ*_*share*_=0.01, the average quality of the population (4.07, Table 2 when several GA runs are averaged) is close to the value without fitness sharing (3.88, Table 2), and even when the individuals are far away from each other, it means that there are many distant areas of the fitness landscape with better adapted codes than the canonical one. With a larger sharing radius (Fig. 7 c, *σ*_*share*_=0.1), the expansion of the population in different and distant areas is more pronounced, as indicated now by the greater inter-distances, where most of them are in a continuous range between 0.14 and 0.46. It should also be noted that these inter-distances between the evolved codes are lower than the distances of those codes to the canonical one. It means that the canonical code is far from the area where most of the evolved codes are located. Therefore, the fitness landscape seems more like a vast space with a broad transition area to the optimum, in the sense that the results resemble the search of the global minimum in the parabola example of Fig. 3. The difference is that now the space surface can be rugged with many non-deep (in comparison with the depth of the best solutions) local peaks.

### Evolution of non-restrictive codes

**Fig. 8:**
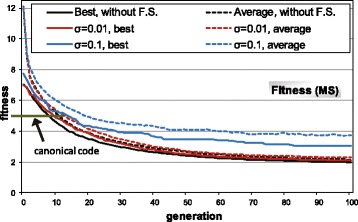
MS evolution through generations with the unrestrictive codes. All graphs are an average of 10 independent GA runs, without fitness sharing and fitness sharing with two values for the parameter *σ*_*share*_ (sharing radius). The horizontal line shows the canonical code MS value

We repeated the analysis with the model of unrestrictive codes. The number of possible codes is now close to 10^84^ [9] with respect to the previous case (2.43·10^18^ possible restrictive codes). The huge increase in the possible codes will allow to check whether the same conclusions are obtained with respect to the previous case. Once more, Fig. 8 shows the evolution across 100 generations of the GA with the same setup as in the previous case, except that now both genetic operators (swap and mutation) are used. The evolution graphs correspond again to an average of 10 independent runs of the GA with different initial populations.

The same conclusions can be obtained with respect to the restrictive codes case. Now all the evolutions are more continuous, given the larger possibilities of codes and landscape areas to explore. The evolution without fitness sharing obtains a possible best code that is more optimized with respect to the use of restrictive codes. This is because the GA has more possibilities to search for adaptive codes against base mutations. Taking into account the average MS value of random codes and the best value of the GA runs, the p.d.m. value is 67% [23], meaning that the canonical code is less optimized (considering unrestrictive codes) with respect to the previous case (p.d.m. value 71% with restrictive codes). This is logical, since the restrictive codes impose constraints to obtain optimized codes. Table 3 summarizes the statistic information regarding the evolutions of Fig. 8, which shows a slight increase in the variability of the final results with larger values of the parameter *σ*_*share*_.

**Table 3:** Summary of statistics regarding Fig. 8. The values are an average of 10 independent runs of the GA

		Average	Standard
		final value	deviation
Without fitness sharing	Best fitness	1.97	0.02
	Average fitness	2.16	0.03
Fitness sharing, *σ*_*share*_=0.01	Best fitness	2.07	0.05
	Average fitness	2.25	0.05
Fitness sharing, *σ*_*share*_=0.1	Best fitness	3.11	0.25
	Average fitness	3.80	0.25

In the analysis of a variety of the best non-restrictive codes, the amino acids that appear in most of the codons have an intermediate value of polar requirement. This helps to minimize the MS error, when most of the mutation changes are among the intermediate values. It is the same idea expressed by Di Giulio [18], when the author used a simulating annealing algorithm to find optimized non-restrictive codes. As the author stated “by maximizing the number of synonymous changes in the code, it is reasonable to suppose that the objective function value is taken towards the absolute minimum” [18]. For example, the author obtained a best code with 42 synonymous codons to one amino acid, one codon to the remaining nineteen amino acids, in addition to the three codons for the termination meaning. However, in our case, the global search of the genetic algorithm finds optimized codes with a more balanced number of codes per amino acid, where only the amino acids with extreme values of polar requirement are associated with only one codon.

**Fig. 9:**
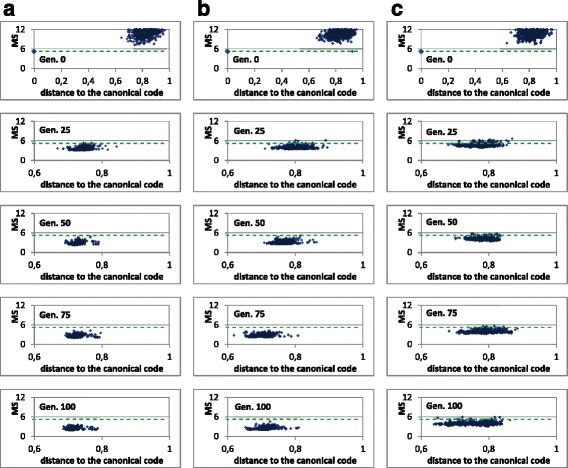
MS value of the genetic population codes vs. their distances to the canonical code. The same graph is shown in different generations of a GA run, in 3 cases with the unrestrictive codes: **a** without fitness sharing, **b** fitness sharing with *σ*_*share*_=0.01 and **c** fitness sharing with *σ*_*share*_=0.1. The canonical code was introduced in the initial population in the 3 cases (point with larger size). The green dashed line represents the MS value of the canonical genetic code

Figure 9 illustrates, in different generations of the evolutionary algorithm, the MS value of each code (*y*-axis) vs. the distance of each encoded code in the population to the canonical code (*x*-axis). The canonical genetic code was inserted in the initial population. Throughout the evolutionary process, the whole population is moving towards better values of adaptability (lower MS values). In fact, in the three cases considered, without fitness sharing (Fig. 9 a) and with fitness sharing with two sharing radius (Fig. 9 b and c), the average value of the population has a better value than the MS value of the canonical code (5.19), as previously seen also in Fig. 8. As in the previous case with restrictive codes, the canonical code does not survive in the first generations of the evolutionary process, even with the use of fitness sharing. This fact, together with the large distances of the optimized solutions at final generations with respect to the canonical code, are evidences for supporting the finding that, using the unrestrictive codes, the canonical code is clearly not located in a broad and deep peak; that is, the canonical code is not in a niche or promising area where some of the solutions of the genetic population could be refining their search. Again, the analyses with different values of *σ*_*share*_, in an ample range between 0.001 and 0.5, present the same evidence, so these are not shown in the Figures.

**Fig. 10:**
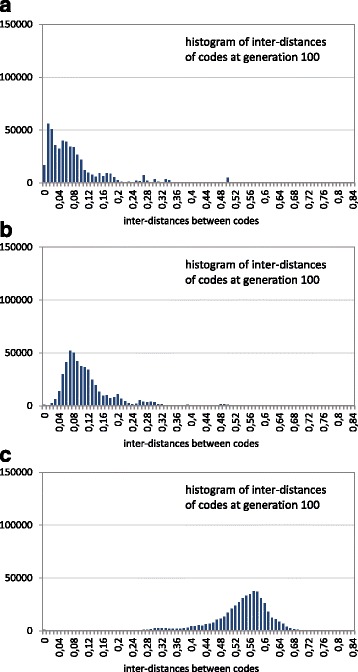
Histogram of code inter-distances of the GA final population using the unrestrictive codes. The histogram corresponds to the final population (generation 100) in a GA run with three cases: **a** without fitness sharing, **b** fitness sharing with *σ*_*share*_=0.01 and **c** fitness sharing with *σ*_*share*_=0.1

The same previous analysis considering the inter-distances of the final population was performed in order to detect how far such final optimized solutions are, that is, how many areas are being simultaneously searched even when the evolutionary process is ended. Note that the inter-distances can have greater values with respect to the previous case with restrictive codes, since the differences between the extreme values of the polar requirement can occur more times with the unrestrictive codes (the same amino acids with those extreme values can be encoded by many codons). Figure 10 shows that the codes are further away from each other as the sharing radius becomes larger. In Fig. 10 a, without fitness sharing, some individuals correspond with the best solution (inter-distance 0). When fitness sharing is used, there are very few solutions corresponding with the best code. Figure 10 b, using a low sharing radius (*σ*_*share*_=0.01), shows that the inter-distances are slightly larger, therefore the individuals are further away from each other with respect to the case without fitness sharing, that is, the final population is more expanded through the fitness landscape. With a larger sharing radius (Fig. 10 c, *σ*_*share*_=0.1), the expansion of the population through the fitness landscape is clearly more pronounced, as can be seen with the high increase of the inter-distances, most of them being between 0.27 and 0.69. Even with the expansion of the population in distant areas of the fitness landscape, the average quality is better than that of the canonical code (Fig. 8). Consequently, the conclusion is again that the fitness landscape does not present a multimodal nature, since there is no niching or clustering effect of the population in promising areas.

### Introduction of transition/transversion and translational biases

Previous works [10, 23] have demonstrated that the canonical code has better adaptability levels when the code fitness takes into account the different probabilities of transition and transversion mutations, together with the mistranslation of mRNA, which implies different probabilities of mutations in the three codon bases. For example, using tMS (“Methods” section), in [23], with the restrictive codes case, the p.d.m is 84%. The comparison of this value with respect to the use of MS as fitness (p.d.m=71%) means that the canonical genetic code is better adapted when the tMS calculation is taken into account. Consequently, a test was conducted to check whether these considerations in the tMS error measure imply a change in the fitness surface nature.

**Fig. 11:**
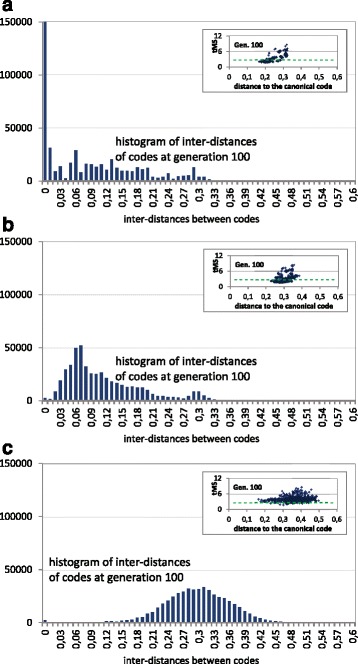
Histogram of code inter-distances of the final population and using the restrictive codes with tMS. The insets show the tMS value of the hypothetical codes of the genetic population vs. their distances to the canonical code. The *green dashed line* represents the tMS value of the canonical genetic code. Each subfigure corresponds with the histograms of inter-distances of the final populations. The graphs correspond to the final population (generation 100) of a GA run in 3 cases: **a** without fitness sharing, **b** fitness sharing with *σ*_*share*_=0.01 and **c** fitness sharing with *σ*_*share*_=0.1

Figure 11 summarizes the results with the restrictive codes. It includes the analysis of tMS versus distances to the canonical genetic code, only at the final generation of the GA runs, in addition to the histograms of inter-distances of the final population from the evolutionary algorithm. As in the previous cases, a GA run without fitness sharing (Fig. 11 a) and with fitness sharing (Figures 11 b and 11 c) were considered, and the canonical code was introduced in the initial population in the different GA runs.

The comparison with the previous cases using MS as objective to minimize (Figs. 5 and 7) shows that the inter-distances are quite similar when tMS is used. For example, the comparison between the histograms in Figs. 11 and 7 shows how the inter-distances are similar using tMS in the three cases (with and without fitness sharing) without significant variations. However, the distances of each code with respect to the canonical code are now lower. Using tMS these distances to the canonical code are between 0.16 and 0.5, with a larger interval with greater values of *σ*_*share*_. In the case of using MS, these distances were between 0.3 and 0.55 (Fig. 5), also with greater intervals with larger values of *σ*_*share*_. This means that the incorporation of tMS implies that the optimized codes are closer to the canonical code, showing again better adaptability of the canonical code considering tMS. However, even with the improvement of adaptability when considering tMS, the final solutions are far from the canonical code while the population is extended across an ample area of the fitness landscape, and without any presence of clear niches in the landscape.

**Fig. 12:**
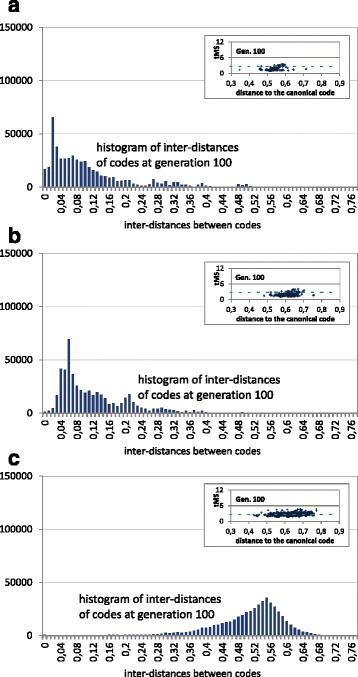
Histogram of code inter-distances of the final population and using the unrestrictive codes with tMS. The insets show the tMS value of the hypothetical codes of the genetic population vs. their distances to the canonical code. The green dashed line represents the tMS value of the canonical genetic code. Each subfigure corresponds with the histograms of inter-distances of the final populations. The graphs correspond to the final population (generation 100) of a GA run in 3 cases: **a** without fitness sharing, **b** fitness sharing with *σ*_*share*_=0.01 and **c** fitness sharing with *σ*_*share*_=0.1

Figure 12 illustrates the same analysis with the unrestritive codes. The histograms of Figs. 12 (tMS) and 10 (MS) are again very similar. However, there is a clear difference in the comparison of the distances of the individuals of the final population with respect to the canonical code (insets of Figs. 12 and 9). With tMS, the distances are between 0.4 and 0.8, whereas with MS those distances are approximately between 0.65 and 0.85, in both cases (tMS and MS) with a tendency of larger intervals with larger values of *σ*_*share*_. Thus, although the final codes are similarly extended in the search space using tMS or MS, the codes of the final population are closer to the canonical code using tMS, which is a further evidence of the better optimization of the code considering tMS.

## Discussion

An adapted genetic algorithm for searching for possible alternative genetic codes better adapted than the canonical code was used in this work. The results of the GA clearly indicate that there are many alternative codes with a better adaptability than the canonical one, being the canonical code relatively far from the best possible adapted one. However, another question to explore is whether the canonical code is in an area corresponding or not to a deep local minimum in relation to the vast fitness landscape, which has been continuously discussed in previous works. This is the issue that has been explored in this work.

As indicated in the Introduction, this is not the same as discerning whether the canonical code is in a local peak (or close to it) regarding its immediate neighborhood. As stated by Novozhilov et al. “The fitness landscape of code evolution appears to be extremely rugged, containing numerous peaks with a broad distribution of heights, and the standard code is relatively unremarkable, being located on the slope of a moderate height peak” [21] (in their case, the authors weighted differently transition and transversion biases in the codon bases). Instead, our aim was to discern about the nature of the vast fitness landscape, inspecting whether it is a multimodal landscape with clear niches, with separated and deep broad MS peaks, together with the location of the canonical code in such a huge landscape.

As explained, due to the huge dimensionality of the search landscape, the incorporation of the fitness sharing technique in the evolutionary algorithm helps to determine and visualize whether the alternative codes, as well as the canonical code, are in an area corresponding to a deep local minimum.

The limitations of the fitness sharing technique are well-known, and basically entail that: i) Setting the dissimilarity threshold (sharing radius) requires a priori knowledge of how far apart the optima are and ii) The complexity per generation is *O*(*N* ^2^) as a consequence of the distance calculations. Even with the complexity drawback, the technique was used because it helps to visualize the fitness landscape (the possible multimodal nature of the landscape). In order to overcome the first limitation, an analysis with different sharing radii was performed.

In the discussion about the location of the canonical code, the general idea is to consider that the canonical code was trapped in a local minimum. For example, according to Crick “there is no reason to believe, however, that the present code is the best possible [...]. Instead, it may be frozen at a local minimum which it has reached by a rather random path” [1]. Or, as Knight et al. state “although search algorithms can sample billions of different codes, evolution is unlikely to have had similar opportunity given the extreme cost of changing an already functional code, and so we might either expect the code to be trapped at a local, rather than global, optimum” [34].

The engineering approach for obtaining better adapted codes was used in our work, since a computational search method was employed for obtaining possible better adapted codes than the canonical one. On the other hand, in the statistical approach, its main idea is that the standard code minimizes hydrophobicity errors far more than can be explained by chance. We are not in disagreement with this idea since the code is more optimized with respect to random codes, but at the same time our analysis indicates that the code is clearly far from possible optimal codes. Some authors within the statistical approach, such as Freeland [29], have argued against the engineering approach bases because the criticisms about the fact that the canonical code is far from optimal “overlook the fact that we know very little about the connectivity or accessibility of local and global optima for patterns of codon assignment”. Nevertheless, the present study helps to visualize the possible formation of local minima in the fitness landscape, indicating that the canonical genetic code is not in an area of a broad and deep local minimum, since it is not captured in a niche with the fitness sharing technique.

Note that our objective was not to explain the possible evolutionary paths to the canonical code. Our objective was only to locate the canonical genetic code in the fitness landscape when possible hypothetical codes are considered, from an scenario of hypothetical codes without restrictions in the assignments to the consideration of restrictive hypothetical codes with the same codon set structure as the canonical genetic code. Moreover, the fitness landscape depends also on the definition of the fitness function, that is, how the adaptability level of a code is measured. We considered the basic property, the polar requirement of amino acids, the most used one in previous studies, in order to define the fitness landscape, since it is the main property for the folding of a protein and consequently to define its protein function. In this sense, Freeland is correct in the criticism “The evolutionary similarity of amino acids (meaning their substitutability within proteins) is unlikely to be perfectly represented by a single physiochemical measure (e.g. polar requirement) or indeed by any simple combination of two or three such indices” [29]. This analysis with other amino acid properties has been performed in several previous works [12, 23, 25, 35]. For example, when different amino acid properties (hydropathy index, isoelectric point and molecular volume, the same as those used by Haig and Hurst [12]) were employed, the results in [23] indicated that polar requirement is the property that provides the most significant evidence of error minimization. Thus, we have included only the most meaningful analysis with the polar requirement property.

## Conclusions

The conclusions obtained in this study can be briefly summarized as follows:

1. The canonical code is better optimized with respect to random codes, when the effects of mutations are taken into account within the error minimization theory. However, the GA search indicates that the canonical code is far from the best possible codes.

2. When the fitness sharing technique is introduced in the GA search, it indicates that there are no clear niches in the vast fitness landscape, that is, localized areas of high fitness (low MS) separated by barriers of low fitness (high MS values). This is not in contradiction with the rugged nature of the fitness landscape. For example, Novozhilov et al. stated that a huge number of taller fitness peaks (with respect to the peak in which the canonical code is situated) exist in the landscape [21]. On the contrary, the results in this study denote that there are many connected areas (not clearly separated) with higher fitness than the canonical code, as inferred from the results of the distances of the optimized codes with respect to the canonical code and the inter-distances between the optimized codes of the GA final populations. That is, the fitness landscape is rugged but does not have a multimodal nature with clear and deep niches separated by fitness barriers. This explains why any search algorithm easily discovers better adapted codes than the canonical one.

Even when the canonical code shows better adaptability levels when the code fitness takes into account different weights for transition and transversion errors in the different codon bases, together with the mistranslational error weights in the three codon bases [10, 23], the same conclusion about the multimodal nature of the fitness landscape is obtained when such biases are considered.

3. The canonical code is clearly far away from those areas of higher fitness in the landscape. Given the non-presence of deep local minima in the landscape, although the code could evolve and different forces could shape its structure, the fitness landscape nature considered in the minimization theory does not explain why the canonical code ended its evolution in a location which is not an area of a localized deep MS minimum of the huge fitness landscape.

## Abbreviations

GA: Genetic algorithm
MS: Mean squared
P.D.M: Percentage distance minimization
tMS: Mean squared considering transition/transversion and translational biases
